# First report on Vitamin B_9_ production including quantitative analysis of its vitamers in the yeast *Scheffersomyces stipitis*

**DOI:** 10.1186/s13068-022-02194-y

**Published:** 2022-09-19

**Authors:** Luca Mastella, Vittorio G. Senatore, Lorenzo Guzzetti, Martina Coppolino, Luca Campone, Massimo Labra, Tiziana Beltrani, Paola Branduardi

**Affiliations:** 1 Department of Biotechnology and Biosciences, University of Milano Bicocca, Piazza della Scienza,2, 20126 Milan, Italy; 2 Laboratory for Resources Valorization (RISE), Department for Sustainability, ENEA- Italian National Agency for New Technologies, Energy and Sustainable Economic Development, S. Maria di Galeria, Rome, Italy

**Keywords:** Nutraceuticals, Biorefinery, 5MTHF, THF, *Scheffersomyces stipitis*

## Abstract

**Background:**

The demand for naturally derived products is continuously growing. Nutraceuticals such as pre- and post-biotics, antioxidants and vitamins are prominent examples in this scenario, but many of them are mainly produced by chemical synthesis. The global folate market is expected to register a CAGR of 5.3% from 2019 to 2024 and reach USD 1.02 billion by the end of 2024. Vitamin B_9_, commonly known as folate, is an essential micronutrient for humans. Acting as a cofactor in one-carbon transfer reactions, it is involved in many biochemical pathways, among which the synthesis of nucleotides and amino acids. In addition to plants, many microorganisms can naturally produce it, and this can pave the way for establishing production processes. In this work, we explored the use of *Scheffersomyces stipitis* for the production of natural vitamin B_9_ by microbial fermentation as a sustainable alternative to chemical synthesis.

**Results:**

Glucose and xylose are the main sugars released during the pretreatment and hydrolysis processes of several residual lignocellulosic biomasses (such as corn stover, wheat straw or bagasse). We optimized the growth conditions in minimal medium formulated with these sugars and investigated the key role of oxygenation and nitrogen source on folate production. Vitamin B_9_ production was first assessed in shake flasks and then in bioreactor, obtaining a folate production up to 3.7 ± 0.07 mg/L, which to date is the highest found in literature when considering wild type microorganisms. Moreover, the production of folate was almost entirely shifted toward reduced vitamers, which are those metabolically active for humans.

**Conclusions:**

For the first time, the non-*Saccharomyces* yeast *S. stipitis* was used to produce folate. The results confirm its potential as a microbial cell factory for folate production, which can be also improved both by genetic engineering strategies and by fine-tuning the fermentation conditions and nutrient requirements.

**Supplementary Information:**

The online version contains supplementary material available at 10.1186/s13068-022-02194-y.

## Background

Stephen DeFelici introduced the term “nutraceuticals” to categorize a wide range of molecules with a claimed medical or health benefit [1]. Over the past few years, a large number of new nutraceuticals has been launched on the food and pharmaceutical market, among which vitamins represent an important category. In a scenario of environmental sustainability, biotechnological production of vitamins is starting to replace the chemical synthesis [2], with interesting examples including vitamins B_2_, B_12_, C, and K [3–6]. However, a biotechnological process for the large-scale production of vitamin B_9_ (or folic acid, FA) has not been implemented yet, and the market still relies on its chemical production, which requires unsustainable, petroleum-based reagents [7].

Folate is the term encompassing the different natural forms of the water-soluble vitamin B_9_. All the vitamers share a common structure consisting in a pteridine ring linked to a molecule of *para*-aminobenzoic acid (*p*ABA) by a methylene bridge, and one or more glutamyl residues. Humans depend on an adequate and constant intake of this essential nutrition component, as it is a central cofactor in many metabolic reactions required for biosynthetic and cellular processes, such as methylation reactions and the synthesis of DNA, RNA and proteins [8]. Indeed, the National Institute of Health (NIH) suggests a Recommended Dietary Allowance (RDA) of 400 μg dietary folate equivalents (DFE) for adults; the European Union (EU) suggests an RDA of 250 μg DFR. A higher intake (600–1000 μg DFE) is advised for pregnant women [9].

Nowadays, the most widely used vitamer for the formulation of food supplements is FA; however, this form is not bioactive, since humans require the activity of dihydrofolate reductase (DHFR) in the enterocytes [10] to reduce FA to one of its active forms (e.g., tetrahydrofolate (THF) and 5-methyl-THF (5MTHF)). The low reaction rate of DHFR [7] limits the absorption of FA, which might lead to its accumulation in the bloodstream. This could mask a vitamin B_12_ deficiency and increase the risk of developing prostate and colorectal cancer [11, 12]. Food supplements enriched with natural forms of folate—which contain the bioactive vitamers—could prevent this issue. Only plants and a few microorganisms possess the entire pathway for the de novo biosynthesis of these bioactive molecules: therefore, the development of more environmentally friendly processes using microorganisms to produce natural folate is becoming crucial [7].

Many studies have focused on the use of lactic acid bacteria—such as *Lactococcus (Lc.) lactis* and *Streptococcus thermophilus*—as potential producers, since they are commonly used in fermented dairy goods and thus can be applied to fortify such products [13]. However, lactic acid bacteria are well-known for their limitations and challenges on an industrial scale, among which the requirement of complex nutritional media for normal growth and a still difficult optimization and control of the metabolic activities [14]. Yeasts, on the other hand, are in general more robust than bacteria and less subject to contaminations, and thus are generally preferred for large-scale fermentations [15, 16]. Conveniently, different *Saccharomyces cerevisiae* strains and other yeasts such as *Metschnikowia lochheadii*, *Debaryomyces melissophilus* and *Debaryomyces vanrijii* have shown to be promising for folate synthesis, with production ranging from 40 to 140 μg/g of cell dry weight [17].

**Scheme 1:**
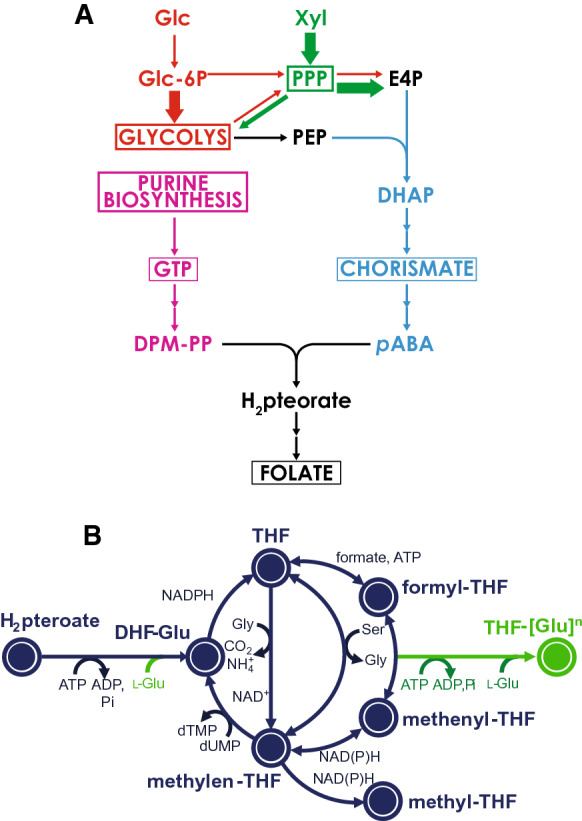
*De novo* folate biosynthesis pathway in *S. stipitis*. **A** Two main building blocks of folate derive from GTP (highlighted in purple) and pABA (highlighted in blue). GTP is produced in the purine biosynthesis pathway. pABA derives from chorismate, the last metabolite produced in the shikimate pathway which has E4P and PEP as precursors (highlighted in black). The first intermediate is produced in the PPP pathway (in green), while the second metabolite is an important molecule mostly produced through glycolysis (in red). The arrow thickness suggests a different flux distribution through the pathways depending on the carbon source. **B** Representation of the pathway in which the different vitamers of vitamin B_9_ are produced. Glc = glucose; Xyl = xylose; Glc-6P = glucose 6-phosphate; PPP = pentose phosphate pathway; E4P = erythrose 4-phospate; PEP = phosphoenolpyruvate; DHAP = 3-deoxy-d-arabino-heptulosonate-7-phosphate; DPM-PP = (7,8-dihydropterin-6-yl)methyl diphosphate; pABA = para-aminobenzoic acid; H_2_-pteroate = dihydropteroate; THF = tetrahydrofolate; DHF = dihydrofolate; Gly = glycine; Ser = serine; Glu = glutamate

In this work, *Scheffersomyces stipitis* was selected as a new potential yeast platform for vitamin B_9_ production. *S. stipitis* is a Crabtree negative yeast [18], which means the onset of ethanol production is not correlated with the sugar concentration: this aspect is interesting from an industrial point of view, since the most commonly used yeast (i.e., *Saccharomyces cerevisiae*) requires more complex fed-batch fermentations to avoid ethanol production. Moreover, *S. stipitis* has the ability to grow on a wide panel of sugars and oligomers, containing both hexose and pentose sugars [19]. The most interesting characteristic for this study is its high flux through the pentose phosphate pathway [20], which is relevant for the production of Vitamin B_9_, since there is a higher availability of erythrose 4-phosphate (E4P), a precursor of one of the moieties of folate (Scheme 1).

In this work, we first optimized the growth of *S. stipitis* on minimal Verduyn medium formulated with 20 g/L of glucose or xylose as carbon and energy source. We assessed the key role of salts, oxygen and nitrogen source to support the growth and allow the complete consumption of the carbon source. Furthermore, we assessed *S. stipitis* growth on optimized Verduyn synthetic medium formulated with a mixture of glucose and xylose, considering the potential to use this strain for the valorization of residual lignocellulosic biomasses. We obtained a robust folate production of 3.72 ± 0.07 mg/L (192.7 ± 46.9 μg/gCDW), both in baffled flasks and bioreactor, which to the best of our knowledge is the highest found in literature when considering wild type microorganisms.

## Results

### Growth optimization on glucose or xylose as carbon source

**Fig. 1:**
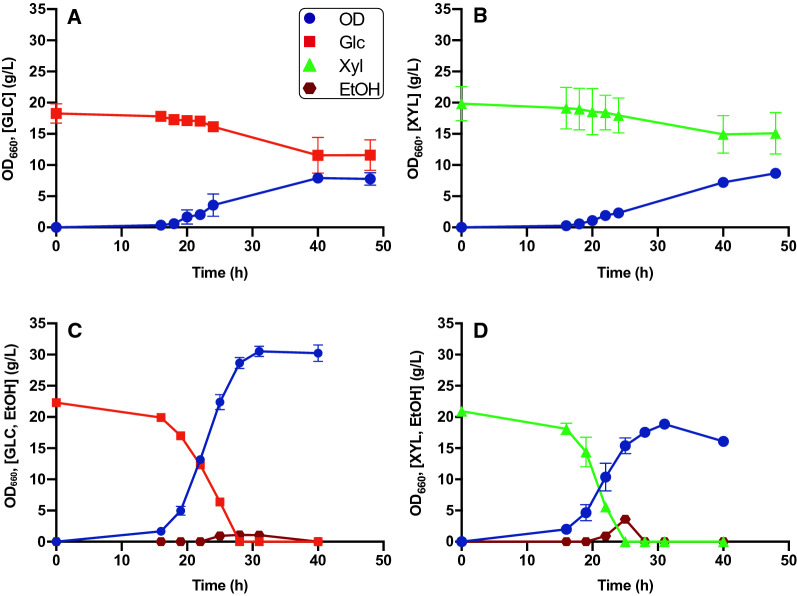
*S. stipitis* fermentation profiles in the presence of glucose or xylose. The graphs show growth in: **A** Verduyn 20 g/L glucose. **B** Verduyn 20 g/L xylose. **C** Verduyn-S2 (20 g/L glucose) in baffled flasks. **D** Verduyn-S2U (20 g/L xylose) in baffled flask. Values are the mean ± standard deviation of three independent experiments

*S. stipitis* was grown on Verduyn minimal medium, using 20 g/L glucose or xylose as carbon source. Under these conditions, only a small consumption of the carbon source was observed (around 4 g/L), both in presence of glucose or xylose (Fig. 1A, B); the result is not entirely unexpected, as Verduyn is a medium optimized for *Saccharomyces cerevisiae*, and it is likely that *S. stipitis* exhibits different demands in terms of nutrients needed for growth.

In line with this hypothesis, by doubling the salts present in the medium (Verduyn-S), a complete consumption of glucose was observed (Additional file 2: Figure S1A). Considering the rather slow consumption of the carbon source and the low biomass yield (Additional file 2: Figure S1A), it was hypothesized that growth might be limited by the availability of O_2_, as reported by Silva, et al. 2012 [21].

Increasing the medium oxygenation by changing the ratio between media and flask volume, cells reached higher OD and biomass yield, despite an incomplete consumption of the carbon source and EtOH production (Additional file 2: Figure S1B). To minimize that, the kinetics were repeated in baffled flasks that allow a greater oxygenation than the traditional ones [22]. Moreover, all the nutrients present in Verduyn-S medium have been doubled (Verduyn-S2), except for the carbon source, and with this formulation *S. stipitis* was able to consume all the glucose present in the medium, reaching 30.5 OD with a substrate consumption rate of 1.73 g/Lh (Fig. 1C). The biomass yield, however, was lower than in the previous condition (1.36 OD/g), due to the production of EtOH, which is around 1 g/L, probably due to the high amount of biomass; once again, oxygen appears to be the most difficult parameter to control in shake flask fermentations.

Likewise, growth on xylose 20 g/L was investigated. The information obtained from the growth optimization of *S. stipitis* in Verduyn with glucose was used to optimize its growth on xylose. The kinetics were performed in baffled flasks using Verduyn-S2 medium; in these conditions, the strain exhibited a better growth, compared with the initial tested condition (Fig. 1B), in spite of an incomplete xylose consumption (Additional file 2: Figure S2).

To obtain the complete consumption of the carbon source, we tested the possibility to repeat the kinetics but changing the nitrogen source from ammonium sulfate to urea, as this formulation is often used for *S. stipitis* [21, 23]. To keep the same amount of nitrogen, 9.2 g/L urea were added to the medium (Verduyn-S2U). As shown in Fig. 1D, urea allowed the complete consumption of 20 g/L xylose with a consumption rate of 2.40 g/Lh, and the biomass reached a final OD of 20.9. However, given the higher growth rate, a production of ethanol (3.59 g/L) was observed, which could explain the lower biomass yield (0.91 OD/g). As observed previously, oxygen appears as the limiting factor for the growth in flasks.

### Growth optimization on media formulated with glucose and xylose as carbon sources

**Fig. 2:**
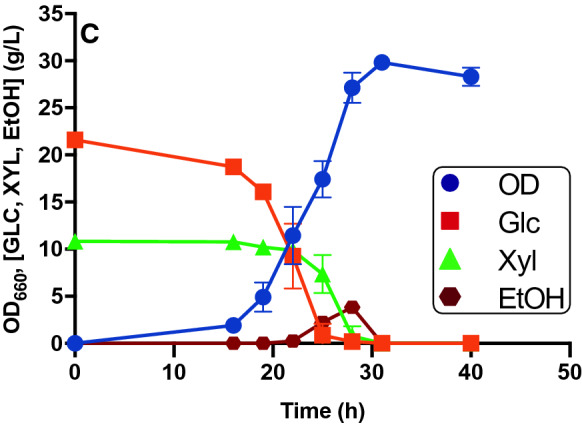
*S. stipitis* fermentation profile in the presence of glucose and xylose. The graph shows yeast growth on Verduyn-S2U Glc 20 g/L, Xyl 10 g/L in baffled flasks. Values are the mean ± standard deviation of three independent experiments

In literature there are several studies on the composition of different lignocellulosic residual biomasses, see as examples [24, 25]. As reported, in corn stover, wheat straw or bagasse and others, the glucose concentration is often double with respect to xylose. With the final aim to use *S. stipitis* to valorize different residual biomasses for vitamin B_9_ production, we opted to simulate that sugar composition, formulating a minimal medium that contains glucose and xylose in a 2:1 ratio. In preliminary studies (Additional file 2: Figure S3, A, B), *S. stipitis* showed a behavior similar to what we observed when grown on glucose or xylose alone. Since previous results showed that Verduyn-S2 medium is required to allow the consumption of 20 g/L of glucose, and since the use of urea as an alternative nitrogen source allows the complete consumption of 20 g/L of xylose, the kinetics were repeated in baffled flasks using Verduyn-S2U. We could confirm that this medium is able to support the complete consumption of the carbon sources. We also observed that xylose consumption starts only when the concentration of glucose present in the medium goes below 10 g/L. Indeed, as reported in literature, *S. stipitis* has two transport systems for sugars; one at high and one at low affinity, which operate simultaneously. Glucose inhibits xylose transport through the high-affinity system, but competes with xylose for the low-affinity transport [24]. Considering the data obtained with single sugars, it might be argued that the preference for glucose when present at high concentration is explained by the difference in the biomass accumulation (30.5 OD on glucose and 20.9 OD on xylose, see again Fig. 1C, D). This means that *S. stipitis*, although it would seem to consume glucose more slowly, prefers this sugar, because it allows a faster growth and a higher biomass yield. In Verduyn-S2U with 20 g/L of glucose and 10 g/L of xylose cell density reached 29.8 OD, with a yield of 0.92 OD/g (Fig. 2). This low yield can be justified by the production of about 3.8 g/L of EtOH, once again due to insufficient oxygenation.

### Folate production in different growth conditions

The overall production of folate was evaluated in the most promising growth conditions, which are Verduyn-S2 glucose 20 g/L, Verduyn-S2U xylose 20 g/L, and Verduyn-S2U glucose 20 g/L and xylose 10 g/L, all in baffled flasks (see again Figs. 1C, D and 2C). Folate concentration was measured at different time points (25, 28 and 31 h), corresponding to Exponential Phase (EP), Late Exponential Phase (LEP) and Early Stationary Phase (ESP) of growth, respectively. We observed that the overall folate production changes in different growth conditions, the best medium being the one with xylose and urea (1D, 2C).

**Fig. 3:**
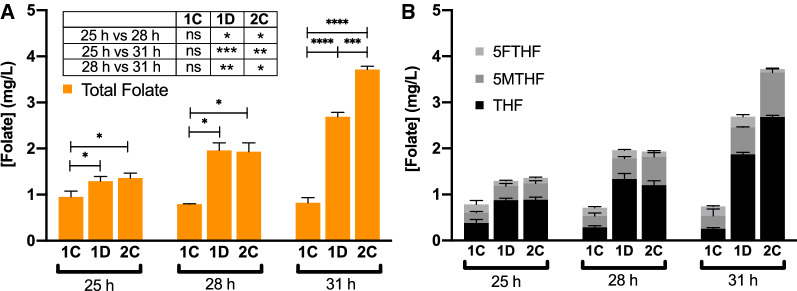
*S. stipitis* folate production and characterization over time in different growth conditions. **A** Total folate production (orange bars). **B** Characterization of the different folate vitamers produced. (1**C)** Kinetics on Verduyn-S2, Glc 20 g/L m/f = 1:5 in baffled flask; (1**D**) Kinetics on Verduyn-S2U, Xyl 20 g/L, m/f = 1:5 in baffled flasks; (2**C**) Kinetics on Verduyn-S2U, Glc 20 g/L, Xyl 10 g/L m/f = 1:5 in baffled flasks. Values are the mean ± standard deviation of three independent experiments. A Two-way ANOVA was performed to analyze the effect of medium composition and growth phase on folate production

Figure 3A shows total folate concentration in the different media. For all the conditions tested, the highest production was obtained in the ESP (31 h); in particular, production reached 0.9 ± 0.12 mg/L on glucose, 2.7 ± 0.10 mg/L on xylose and 3.7 ± 0.07 mg/L on glucose and xylose. Due to the higher sugar concentration, growth on the mixed medium resulted in the highest folate production. Indeed, folate production was 4.5 and 1.4 times higher than in the media containing only glucose and only xylose, respectively.

Figure 3B shows the amount of the different vitamers produced by *S. stipitis* in the conditions tested in this study. After 31 h of fermentation, in conditions 1D and 2C *S. stipitis* was able to produce 1.9 ± 0.05 and 2.7 ± 0.04 mg/L of THF, 0.6 ± 0.01 mg/L and 0.97 ± 0.08 mg/L of 5MTHF, and 0.2 ± 0.05 mg/L and 0.1 ± 0.02 mg/L of 5FTHF, respectively, while FA was not identified in any sample analyzed.

Since the total carbon concentrations provided in the different runs are different, to compare results it is important to calculate fermentation parameters, and here we used the following: product yield (*Υ*_P_), growth rate (*μ*_max_) and duplication time (*T*_D_). On glucose as sole carbon source the cells express the fastest *μ*_max_ (0.34 h^−1^), which is also reflected in the shorter *T*_D_ (2.0 h), but the strain has the lowest *Υ*_P_ = 6∙10^–5^ *g*_P_/*g*_S_. In Verduyn-S2U with xylose as carbon source the situation is reversed, as the strain presents the lowest growth rate (0.28 h^−1^) and a longer duplication time (2.5 h), but the highest *Υ*_P_ = 12.8∙10^–5^ g_P_/g_S_. In the medium with mixed sugars, we registered an intermediate performance, with a growth rate of 0.30 h^−1^ and duplication time of 2.3 h, but with a *Υ*_P_ = 11.5∙10^–5^ g_P_/g_S_, which is close to the performance reached with xylose as sole carbon source.

Considering these observations, the mixed medium with both glucose and xylose was selected for the scale up in bioreactor, to obtain more precise data about growth and folate production by *S. stipitis*, and to assess the possible production performances of this process.

### Growth and folate production in bioreactor

**Fig. 4:**
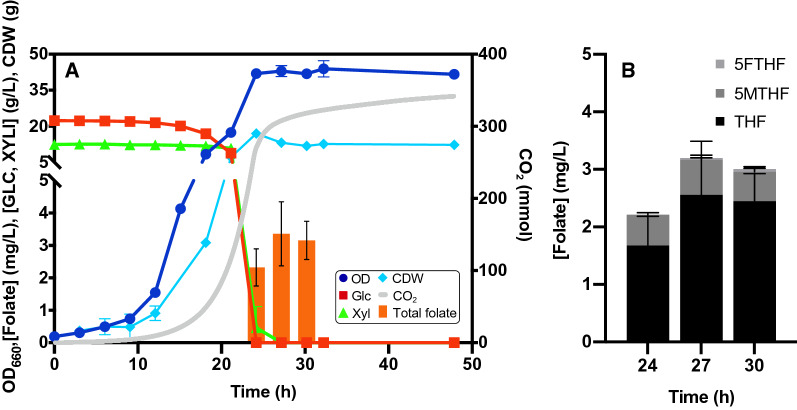
Fermentation of *S. stipitis* in bioreactor on a mixed medium. **A** Fermentation profile and folate productions. **B** Characterization of the different folate vitamers produced. Values are the mean ± standard deviation of independent experiments

To test the robustness of the process in a larger volume while assuring a full aerobic condition and to acquire data for quantitative analysis, we moved to 2 L stirred tank bioreactors, growing the cells in the same medium tested in baffled flasks Verduyn-S2U (20 g/L glucose, 10 g/L xylose); results are shown in Fig. 4A. Thanks to the possibility of monitoring and maintaining the desired settings for pH and dissolved oxygen, we observed the highest cell density of this study (40 OD), with no EtOH production; moreover, *S. stipitis* reached the stationary phase at 24–27 h, showing a reduced fermentation time, with a growth rate of 0.27 h^−1^*.*

Consistently with the results obtained in shake flasks, the peak for folate production was obtained in the ESP (27 h), reaching a concentration of 3.4 ± 0.99 mg/L. The peak production corresponds to 192.7 ± 46.9 μg∙gCDW^−1^, which is reflected in a specific yield of 5.6 ± 1.4 μg∙gCDW^−1^∙gS^−1^. Figure 4B shows the production of the different vitamers. At 27 h (peak production), THF is the main form, with a concentration of 2.6 ± 0.9 mg/L, corresponding to 76% of the total production. 5MTHF is the second most abundant form, reaching a concentration of 0.6 ± 0.04 mg/L (18% of total production). Only a small production of 5FTHF was observed (in a range between 0.04 ± 0.049 and 0.05 ± 0.012 mg/L).

## Discussion

### Growth optimization in shake flasks

In this work we investigated the ability of *S. stipitis* to produce folate, considering various advantages that this yeast can offer. Numerous non-*Saccharomyces* yeasts are known to have evolved in ecological niches different from those of provenance of *S. cerevisiae*: some niches are characteristic for the quality of the substrates, or for the presence of substances usually inhibiting growth, and consequently led to the selection of yeasts with interesting characteristics for growth on complex substrates and to produce new molecules [27], ideal for developing a bioprocess.

Indeed, *S. stipitis* can metabolize different sugars and a wide range of oligomers; it is one of the yeasts with the best ability to ferment xylose to ethanol [19], in combination with a virtual zero production of by-products in anaerobiosis, such as acetate, acetoin, 2,3-butanediol, pentitols and glycerol [18, 28]. Unlike *S. cerevisiae*, *S. stipitis* is a negative Crabtree yeast and it has been shown in several studies [18, 20, 21, 29] that the production of ethanol is triggered when the availability of oxygen becomes limiting, and not by high glucose concentration. This feature can be advantageous in a bioprocess, because it allows to limit the production of ethanol as a carbon sink to maintain the redox balance in aerobic conditions. Another advantage of *S. stipitis* is an increased carbon flux through the Pentose Phosphate Pathway (PPP), which is common in several hemiascomycetes yeasts [30]. Indeed, a profiling analysis of metabolic fluxes in *S. stipitis* showed that 41% of glucose 6-phosphate (G6P) is oxidized through PPP, while in *S. cerevisiae,* the flux is only equal to half of that value [31]; moreover, the percentage of phosphoenolpyruvate (PEP) produced through at least one transketolase (i.e., through the PPP) is around 60% for *S. stipitis*, while for *S. cerevisiae,* it is basically zero, as PEP is virtually synthesized only through glycolysis [32]. This characteristic is very interesting for the synthesis of folate—and more generally for the wide range of compounds that derive from the shikimate pathway—because the low availability of E4P is generally a limiting factor for this type of productions in *S. cerevisiae*; a greater flux through the PPP should be reflected in a greater availability of intermediates and, consequently, in a greater flux through the shikimate pathway. As reported by Gao and colleagues, *S. stipitis* was engineered for shikimate production in aerobic conditions, and the authors commented that in non-limiting oxygen conditions PEP and E4P precursors might be more abundant than in oxygen-limited conditions [33]. Since one of the two main building blocks of folate has pABA as precursor, and since most studies focus on anaerobic fermentation, we decided to optimize the growth on minimal Verduyn medium to sustain the aerobic state, which in turn could promote folate production.

The goal in the first part of the work was to optimize the chemical–physical parameters to maximize growth and production, while at the same time deepening the physiological and metabolic aspects; in fact, defining the composition of the fermentation medium is an important step to increase productivity in bioconversion processes [21].

Additional file 1: Table S2 summarizes the results obtained in shake flask fermentations. Growth optimization on glucose mainly required additional nutrients to sustain a high biomass production, combined with an increased oxygenation to avoid the production of ethanol as a carbon sink.

**Fig. 5:**
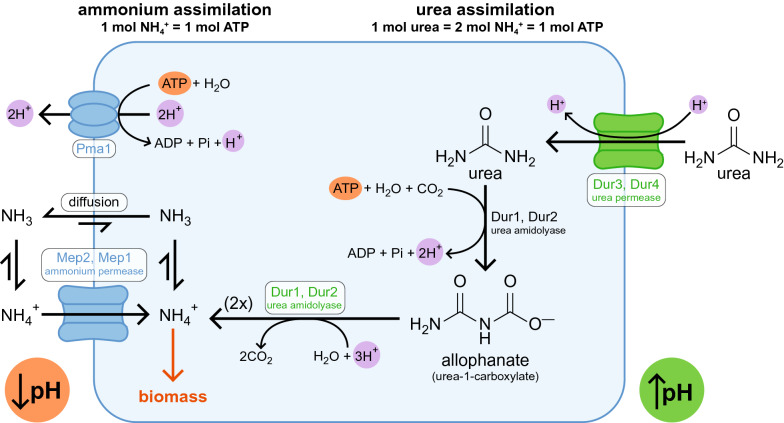
Proposed putative pathway for ammonium and urea uptake in *S. stipitis*. Ammonium (left) is internalized in the cell by facilitated diffusion via ammonium permease; to maintain the potential difference across the plasma membrane the H^+^ -ATPase Pma1 expels a proton, causing acidification of the growth medium. Urea (right) is internalized through a symport with an H^+^ ion (causing an increase in the pH of the growth medium), and it is converted into ammonium via urea amidolyase

The fermentations on xylose under full aerobiosis, however, allowed us to observe an unusual behavior: growth speed and xylose consumption were about three times smaller when compared with the same conditions using glucose as carbon source. The use of urea as an alternative nitrogen source to ammonium sulfate was essential to allow fast growth and the complete consumption of xylose. This behavior is still not clear to us; however, the complete consumption of xylose with urea as a carbon source could be explained by the different energetics of the metabolism when urea is the nitrogen source (Fig. 5). Ammonia (NH_3_) has a pK_a_ of 9.25, so the concentration of ammonium (NH_4_^+^) remains virtually the same for pHs between 3 and 7 [34], indicating that most of the nitrogen supplied as (NH_4_)_2_SO_4_ is present as an ammonium ion. In *S. cerevisiae*, ammonium uptake occurs by facilitated diffusion thanks to an ammonium permease, encoded by *MEP*2 and *MEP1*. The accumulation of intracellular NH_4_^+^ is favored by the negative membrane potential, but the maintenance of homeostasis requires the pumping of a proton to the outside by the H^+^-ATPase Pma1, with a net consumption of 1 ATP for every ammonium ion introduced [34]. Ammonia can permeate the cell membrane by diffusion, but in *S. cerevisiae* the balance is shifted towards the export [34]. Therefore, the uptake of 1 mol of ammonium requires 1 mol of ATP and causes the acidification of the growth medium. The same genes coding for the ammonium permease (*MEP*2, *MEP*1) and for the H^+^-ATPase Pma1 (*PMA*1) are present in *S. stipitis* as well [35], so it is possible to hypothesize that the ammonium uptake mechanism is the same. On the other hand, in *S. stipitis* urea uptake happens through a urea permease, encoded by the *DUR*3 and *DUR*4 genes [19] and the transport occurs via a simport with an H^+^ ion [35]. Since budding yeasts lack the urease enzyme, urea amidolysis (encoded by *DUR*1 and *DUR*2) is required for the metabolism of urea [36]. This bifunctional enzyme first catalyzes an ATP-dependent carboxylation to produce allophanate (urea-1-carboxylate), and subsequently catalyzes its decarboxylation to 2 ammonium ions and CO_2_ [36]. It is interesting to note the elegant stoichiometry of urea uptake and its subsequent transformation reactions (Fig. 5) [37]: the proton imported from the permease and the two protons generated by the carboxylation are all used in the decarboxylation step, with a neutral balance of protons. We then hypothesize that the uptake of urea does not require to be coupled to an active transport of protons. Therefore, the uptake of 1 mol of urea (which produces 2 mol of NH_4_^+^) requires only 1 mol of ATP, half of the energy required for ammonium uptake; moreover, the uptake of urea causes an increase—rather than a decrease—of the medium pH, which might cause less stress to the cells when compared to the growth at a low pH. It is therefore possible to hypothesize that the complete consumption of the carbon source is due to a greater availability of energy. This behavior could also be enhanced by the probable different gene expression with the two nitrogen sources [38]. Moreover, the shift in specificity of xylose reductase (XR) towards NADPH, may play a role in cofactor imbalance in the presence of high concentrations of xylose and oxygen (see Additional file 2: Figure S4 and related comments for further details).

### Folate production

In this work the aerobic growth of *S. stipitis* in Verduyn-based medium formulated with different carbon and nitrogen sources was studied. Folate production was evaluated in the optimized conditions at different time points using a HPLC–UV method to measure four different folate vitamers (THF, 5MTHF, 5FTHF and FA), as this is relevant for assessing their bioactivity. In addition, the occurrence of the target analytes quantified in different growth conditions was qualitatively confirmed by exploiting an HRMS system (high-resolution mass spectrometry) (see Par. 5.5.4). Additional file 1: Table S3 and Additional file 2: Figure S5 report the MS and MSMS spectra of the analytes identified in yeast medium.

Hjortmo and co-workers [39] reported that in *S. cerevisiae* the folate production peak is reached at the early stationary phase. Indeed, we observed a similar behavior with *S. stipitis*, reaching peak production at the ESP, right after the depletion of the carbon source and the rapid consumption of ethanol. To the best of our knowledge, this is the first study investigating *S. stipitis* ability to produce folate. Furthermore, it is also the first study in which the different folate vitamers produced by *S. stipitis* were characterized, showing that this yeast mainly produces vitamers in their reduced forms, such as THF, 5MTHF, and 5FTHF, even though yeast growth is pursued in aerobiosis. Figure 3B shows that medium composition and growth phase significantly affects the level of the different forms produced. While on glucose *S. stipitis* produced more balanced amounts of the various forms, in the media containing xylose and urea the production is biased towards THF.

This behavior was confirmed during bioreactor fermentation. Indeed, we observed a comparable folate production with respect to the same growth medium in shake flasks (2C), suggesting that this process is robust and scalable. More interestingly, the bioreactor setup provided better growth conditions which in turn allowed a shorter fermentation time and an improvement in the productivity, probably due to the lack of ethanol production.

Table 1 shows the highest folate productions found in literature, obtained with different microorganisms, ranging from bacteria, to yeasts, to filamentous fungi. The productions obtained in the present study with *S. stipitis* on minimal medium are among the highest reported in literature, generally being around 10 times higher than the ones obtained with other microorganisms. The only exception is an engineered strain of the filamentous fungi *Ashbya gossypii* [40], which reaches a production of 6.60 mg/L of folate, exceeding approximately 2 times our results. However, in addition to being engineered, *A. gossypii* requires an adenine auxotrophy to sustain such a high production, combined with the supplementation of *p*ABA, with consequences on economics of the possible process.

**Table 1:** Folate productions of different organisms reported in literature

Microorganism	Medium	[Sugars] (g/L)	Production	Refs.
*S. stipitis*	Verduyn-S2	Glc 20	0.95 ± 0.12 mg/L	This study
	Verduyn-S2U	Xyl 20	2.69 ± 0.10 mg/L	This study
	Verduyn-S2U	Glc 20; Xyl 10	3.72 ± 0.07 mg/L	This study
(bioreactor)	Verduyn-S2U	Glc 20; Xyl 10	3.36 ± 0.99 mg/L (192.7 ± 46.9 μg/g_CDW_)	This study
*S. cerevisiae*	YPD	Glc 20	0.25 mg/L	[39]
	Verduyn 2X	Glc 20	0.36 mg/L	[39]
*Y. lipolytica*	YPD	Glc 20	0.3 ± 0.1 μg/gCDW	[41]
*Metschnikowia lochheadii*	CBS modified	Glc 20	90 μg/gCDW	[17]
*E. coli PB25*	M9	Glc 4	0.27 mg/L	[42]
*S. thermophilus*	M17	Lac 5	0.20 mg/L	[43]
*Lc. lactis* subsp. *lactis*	M17	Lac 5	0.29 mg/L	[43]
*Ashbya gossypii*	MA2 + *p*ABA	Glc 10	6.60 mg/L	[40]

Reported productions of this study represent the sum of single vitamers titles

## Conclusions

To the best of our knowledge, this is the first report exploiting the yeast *S. stipitis* for the production of folate. We formulated media considering the main sugars present in lignocellulosic biomasses as carbon sources. The different optimizations in shake flasks allowed the selection of the best condition (Verduyn-S2U medium with 20 g/L of glucose and 10 g/L of xylose, *Υ*_P_ = 11.5∙10^–5^ g_P_/g_S_ and growth rate 0.30 h^−1^) to be repeated and studied in detail in bioreactor. The production obtained in bioreactor in the present study with *S. stipitis* on minimal medium (3.36 ± 0.99 mg/L, or 192.7 ± 46.9 μg/g_CDW_) is the highest reported in literature comparing wild type microorganisms.

Furthermore, the production of folate was found to be predominantly shifted towards vitamers in their reduced forms (2.6 ± 0.9 mg/L of THF, 0.6 ± 0.04 mg/L of 5MTHF and 0.04 ± 0.049 mg/L of 5FTHF), making *S. stipitis* a very promising organism for the nutraceuticals market, since the majority of folate available in food supplements tends to be in the oxidized forms, thus requiring an endogenous reduction step to be metabolically active. The obtained results demonstrate the potential of *S. stipitis* as a microbial cell factory for natural folate production, which can reasonably be further improved by genetic engineering strategies and/or by fine tuning the fermentation conditions and nutrient requirements.

## Materials and methods

### Strain and media composition

The haploid, Crabtree negative yeast *S. stipitis* (culture collection CBS 6054) was used in the experiments. The strain was maintained in 20% (v/v) glycerol at − 80 °C (Master Cell Banks, MCB) after growth in YPD medium composed of (per liter): yeast extract 10 g, tryptone 20 g and glucose 20 g.

Defined synthetic media Verduyn [44] were composed of (per liter): (NH4)_2_SO_4_ 5 g; KH_2_PO_4_ 3 g; MgSO_4_·7H_2_O 0.5 g; trace elements 1X (EDTA 30 mg; ZnSO_4_·7H_2_O 9 mg; CoCl_2_·6H_2_O 0.6 mg; MnCl_2_·4H_2_O 2 mg; CuSO_4_·5H_2_O 0.6 mg; CaCl_2_·2H_2_O 9 mg; FeSO_4_·7H_2_O 6 mg; Na_2_MoO_4_·2H_2_O 0.8 mg; H_3_BO_3_ 2 mg; KI 0.2 mg); vitamins 1X (d-biotin 0.10 mg; calcium d-pantothenate 2 mg; nicotinic acid 2 mg; myo-inositol 50 mg; thiamine hydrochloride 2 mg; pyridoxal hydrochloride 2 mg; *para*-aminobenzoic acid 0.4 mg. Depending on the experiment, the carbon sources used were (per liter): glucose 20 g; xylose 10 g or 20 g; or glucose 20 g and xylose 10 g. The pH of the media was adjusted to 5.5.

Growth optimization first required to double the concentrations of some salts as follows: (NH4)_2_SO_4_, 10 g; KH_2_PO_4_, 6 g; MgSO_4_·7H_2_O, 1 g, obtaining what we called “Verduyn-S” medium. Furthermore, the final growth and production runs were performed in Verduyn-S2, where in addition to salts also trace elements and vitamins present in the Verduyn-S have been doubled; finally, when indicated, ammonium sulfate has been replaced with 9.2 g/L of urea (Verduyn-S2U). Additional file 1: Table S1 summarizes all the media compositions used in this study.

Yeast extract was purchased from Biolife Italia S.r.l., Milan, Italy. All other reagents were purchased from Sigma-Aldrich Co., St Louis, MO, USA.

### Growth conditions in shake flasks

Seed cultures from YPD plates were initially grown overnight in glass tubes in 2 mL YPD; cells were then inoculated for the intermediate inoculum (starting OD 0.2) in 50 mL glass tubes containing 5 mL of the same culture medium used for the subsequent run, and grown for 8 h: this step is important to accustom the cells to the final culture conditions. Cells were then inoculated in 250 mL (baffled) shake flasks (starting OD 0.001), filled with 50 mL of the minimal medium under investigation. All growths were performed in a rotary shaker at 160 rpm and 30 °C.

### Growth conditions in batch bioreactors

For batch fermentations, 2 L stirred tank bioreactors (BIOSTAT® A plus, Sartorius Stedim Biotech GmbH, Goettingen, Germany) equipped with Visiferm DO ECS 225 for pO_2_ measurement and Easyferm Plus K8 200 for pH measurement (both from Hamilton Bonaduz AG, Bonaduz, Switzerland) were used at a working volume of 1 L. The temperature was kept constant at 30 °C and pH was set to 5.5, maintained by automatic addition of 5 M KOH and 5 M HCl. The stirring rate was set to 300 rpm in cascade to maintain the oxygen concentration, which was set to 30% of saturation, to guarantee a completely aerobic condition to the cell culture. Filtered air (pore size 0.2 μm) was continuously sparged through the reactor at a flow rate of 1 vvm. Foam formation was controlled by the addition of an emulsifier agent (Triton 100X, Fisher reagents) and a silicon agent (Polydimetylsiloxane, Sigma-Aldrich). MCB was restreaked in YPD plates. A single colony was taken and grown in YPD liquid medium overnight. From this culture, individual aliquots of 1.3 mL were created and kept in 20% (v/v) glycerol at − 80 °C (Working Cell Banks, WCB).

WCB were thawed and inoculated directly in 50 mL glass tubes containing 5 mL of YPD and incubated for 8 h. Cells were then inoculated in 500 mL shake flasks in 100 mL of minimal medium for the intermediate cultures (starting OD 0.04) and grown overnight. For the inoculum, cells were harvested, washed twice with physiological solution (0.9% NaCl), and used to inoculate the bioreactor (starting OD 0.25). Samples were collected every 3 h until 30 h and then at the end of the fermentation, stopped at 48 h.

### Biomass and metabolites quantification

*S. stipitis* growth was followed by measuring the optical density at 660 nm (OD_660_) (UV-1800; Shimadzu, Kyoto, Japan). For bioreactor experiments cellular dry weight (CDW) was estimated by a correlation with OD_660_, using the equation: CDW = 0.3998OD + 0.1307.

HPLC analyses were performed to quantify the amount of glucose, xylose and ethanol. Prior to analysis, all samples obtained in Par 5.3 were centrifuged (14,000 rpm, 10’, 4 °C) and filtered with 0.2 μm PTFE filters (AISIMÔ CORPORATION CO., LTD). For the analysis of glucose, xylose and ethanol, a Rezex ROA-Organic Acid H^+^ column (00H-0138-KO) 300 × 7.8 mm, 8 μm (Phenomenex, USA) coupled with a precolumn Micro-Guard Cation H + refill cartridges 30 × 4.6 mm, 8 um (Biorad, USA) was injected with 20 μL of sample. The mobile phase was H_2_SO_4_ 0.01 M pumped isocratically at a flow of 0.5 mL/min for 40 min. Column temperature was kept at 40 °C. Separated components were detected by a refractive index detector (RID) and peaks were identified by comparison with known reference standards dissolved in Ultrapure H_2_O (18 MΩ) obtained using a Milli-Q purification system (Millipore, Bedford, USA). Calibration curves were prepared in a range between 20 and 0.625 g/L.

### Total folate determination and quantification

#### Reagents

All reagents were purchased from Merck, Germany, apart from Ultrapure H_2_O (18 MΩ) obtained by a Milli-Q purification system (Millipore, Bedford, USA), THF purchased by Schircks Laboratories, Switzerland, 5MTHF and 5FTHF obtained from US Pharmacopeia, USA. MS grade solvents were obtained from Romil SpA, Italy.

#### Solutions preparation

- Potassium phosphate buffer 1 M (Buffer P) was prepared by adjusting the pH of a solution of KH_2_PO_4_ 1 M to 6.4 by adding H_2_KPO_4_ 1 M; ascorbic acid was then added to a final concentration of 10% (*w/v*); the solution was filter-sterilized and stored at 4 °C.
- Purification of rat serum for polyglutamyl-folate deconjugation was adapted from Patring et al. [45]. Briefly, 1 mL of activated charcoal is added to 10 mL of rat serum and allowed to react for 1 h at 4 °C with constant stirring; the solution was then filtered, divided into 2 mL aliquots, and stored at − 20 °C.

#### Sample preparation

3 mL or 10 mL of culture suspension were collected from shake flasks or bioreactor, respectively. To avoid folate degradation in samples before and during the analysis, 10% of Buffer P 1 M were added to each sample and N_2_ was sparged to replace the oxygen present in the medium; samples were stored at − 80 °C and thawed prior to use.

The measurement of the total folate produced requires the release of intracellular folate directly into the culture supernatant (in which extracellular folates are already present). The protocol was adapted from Patring et al. [45]; intracellular folates were extracted by heating at 100 °C for 15’; after centrifugation (4 °C, 14,000 rpm, 10 min) 1 mL of the supernatant was treated with 50 μL of rat serum at 37 °C for 3 h. After filtration, the sample was used for the analysis. The deconjugation step with rat serum is required to obtain all the present folate in the mono-glutamate forms: this allowed the quantitative analysis at HPLC with the direct standards correlation.

#### Solid-phase extraction

Solid-phase extraction (SPE) was performed using Isolute™ SAX cartridge (Biotage, Sweden) with strong anion-exchange sorbent 500 mg/3 mL. For elution under reduced pressure, a Sigma vacuum manifold was used. The clean-up procedure was as follows: the SPE cartridge was activated and conditioned by sequential elution of 5 mL of MeOH and H_2_O and phosphate buffer 0.1 M (Na_2_HPO_4_) containing 1% ascorbic acid w/v (pH 7) without allowing the column to run dry. Then, samples obtained in Par 5.5.2 were equilibrated to pH 7 by a few drops of NaOH 5 M and then loaded and passed through the cartridge adjusting the vacuum, to keep a constant flow rate of 2–3 drops per second. When all the sample was eluted, the cartridge was washed with 5 mL of phosphate buffer 0.1 M (pH 7) and completely dried. Analytes were eluted into a glass vial with 5 mL of 0.1 M sodium acetate containing 10% (w/v) sodium chloride and 1% (w/v) ascorbic acid. Prior to HPLC analysis, all samples were filtered through a 0.5 μm Millipore filter (Bedford, MA, USA).

#### Lc–Ms separation and qualitative analysis

The identification of folic acid vitamers in the samples was carried out using a Waters ACQUITY UPLC system coupled with a Waters Xevo G2-XS QTof Mass Spectrometer (Waters Corp., Milford, MA, USA). All analytes were separated on a UPLC system equipped with a Zorbax SB-C18 column (100 mm × 2.1 mm, 3.5 µm). The mobile phases were both MS grade H_2_O (A) and MeOH (B), both containing 0.1% formic acid (HCOOH), with gradient elution as follows: 0–2.0 min, 5–10% B; 2.0–17.0 min, 10–35% B; 17.0–18.0 min, 35–95%. After each run the column was washed for 5 min (95% B) and then equilibrated for further 5 min at the initial conditions (5% B) before the next sample injection. Elution was performed at a flow rate of 0.5 mL/min, and the injection volume was 5 μL. The column temperature was set at 30 °C. The Xevo G2-XS QTof Mass Spectrometer, equipped with an ESI source, was used in negative ionization mode to acquire full-scan MS and the spectra were recorded in the range of m/z 100–1000. The source parameters were as follows: electrospray capillary voltage 2.5 kV, source temperature 150 °C, and desolvation temperature 500 °C. The cone and desolvation gas flows were 10 and 1000 L/h, respectively. A scan time of 0.5 s was employed. The cone voltage was set to 60 V, and ramping collision energies ranged from 6 to 30 V to produce abundant ions before detection at the ToF. The mass spectrometer was calibrated with 0.5 M sodium formate and leucine–enkephalin (100 pg/μl) was used as LockMass (m/z 554.2615, 2 kV ionization voltage), which was infused simultaneously with the flow of column at 10 μl/min and acquired for 1 s each 10 s. The base peak chromatograms (BPI) were acquired at low [6] and high [30] energy from which the peaks identification was performed. Folate vitamers identity was confirmed by exploiting analytical standards as reference. Standard solutions were prepared by dissolving 5MTHF, 5FTHF, THF and FA in H_2_O (pH = 9) containing 1% (w/v) sodium ascorbate. The MassLynx software (version 4.2) was used for instrument control, data acquisition and data processing.

#### Quantitative analysis by HPLC–UV

To determine the amount of folic acid vitamers in samples, a 1260 Infinity HPLC system (Agilent Technologies, USA) coupled to a UV detector was exploited. The analytes were separated by a Zorbax SB-C18 column (4.6 × 250 mm, 5 µm) coupled with a precolumn (4.6 × 12.5 mm, 5 µm) both purchased from Agilent Technologies, USA. The mobile phases were: aqueous TBS (Tetrabutylammonium sulfate) 5 mM, K_2_HPO_4_ 3 mM, KH_2_PO_4_ 3 mM pH 7.5 (A) and MeOH (B), with gradient elution as follows: 0–11.0 min, 10–55% B; 11–13 min 55–60%, 13–15 min 55–95% B, 15–18 min 95% B and 18–19 min 95–10% B, 2 and 8 min of equilibration (10% B) was performed before the next sample injection. Elution was performed at a flow rate of 1 mL/min and the injection volume was 100 μL. The column temperature was set at 30 °C. UV spectra were acquired in the range of 190–600 nm and two wavelengths, 280 and 310 nm, were employed for the detection of target analytes. A calibration curve for each analyte was made in a range between 0.1 and 10 μg/mL and regression coefficients were used only if associated with *R*^2^ > 0.98.

#### Fermentation analysis

Specific growth rate (µ_max_) was calculated mathematically by an equation obtained from plotting values of OD vs. time on Excel.

Folate yields on consumed sugars (here ${Y}_{\mathrm{p}}$) were calculated using the following equation:

1 $${Y}_{\mathrm{P}}={F}_{\mathrm{p}}/{\Delta }_{\mathrm{sug}}\cdot 100$$

where ${F}_{\mathrm{p}}$ is the amount of folate produced and ${\Delta }_{\mathrm{sug}}$ the amount of sugar consumed.

Duplication time (${T}_{\mathrm{d}}$) was calculated using the following equation:

2 $${T}_{\mathrm{d}}=\mathrm{ln}2/{\mu }_{\mathrm{max}}$$

Consumption rate (*C*_r_) was calculated using the following equation:

3 $${C}_{\mathrm{r}}={\Delta }_{\mathrm{sug}}/\Delta \mathrm{t}$$

where $\Delta t$ is the time interval corresponding to the exponential phase of the growth kinetics.

#### Statistical analysis

A two-way ANOVA was performed to analyze the effect of medium composition and growth phase on folate production followed by a post hoc Tukey–Kramer test for multiple comparisons. *P* values are represented as follows: *P* > 0.05, ns; *P* ≤ 0.05, *; *P* ≤ 0.01, **; *P* ≤ 0.001, ***; *P* ≤ 0.0001, ****.

## Supplementary Information

**Additional file 1: Supplementary tables. Table S1.** Composition of the different synthetic media used in this study. **Table S2.** Growth conditions, biomass yields and folate production on minimal Verduyn medium. **Table S3.** LC/MS based qualitative determination of folic acid vitamers in *S. stipitis* samples in negative ion current.

**Additional file 2: Supplementary figures. Figure S1. **Fermentation profile on **(A) **Verduyn-S 20 g/L glucose m/f 1:5 and **(B)** Verduyn-S 20 g/L glucose in baffled flasks. **Figure S2.** Fermentation profile on **(A) **Verduyn-S2 20 g/L xylose in baffled flasks. **Figure S3.** Fermentation profile on **(A) **Verduyn 20 g/L glucose + 10 g/L xylose in baffled flasks and **(B)** Verduyn-S2 20 g/L glucose + 10 g/L xylose in baffled flasks. **Figure S4. **Cofactor imbalance for xylose assimilation under different oxygen conditions. **Figure S5.** EIC (Extracted Ion Chromatogram) in negative ion current of a representative sample (in brown) showing the occurrence of the three reduced vitamers and the absence of folic acid compared with the reference standards (in black).

## Data Availability

All data generated or analyzed during this study are included in this published article and its supplementary information file.

## Abbreviations

DFE: Dietary folate equivalents
NIH: National institutes of health
OD: Optical density
pABA: Para amino benzoic acid
RDA: Recommended dietary allowance
DHFR: Dihydrofolate reductase
rpm: Revolutions per minute
FA: Folic acid
THF: Tetrahydrofolic acid
5MTHF: 5-Methyl-tetrahydrofolic acid
5FTHF: 5-Formyl-tetrahydrofolic acid
E4P: Erythrose 4-Phosphate
MCB: Master cell bank
WCB: Working cell bank
CDW: Cellular dry weight
SPE: Solid-phase extraction
EP: Exponential phase
LEP: Late exponential phase
ESP: Early stationary phase
PPP: Pentose phosphate pathway
G6P: Glucose 6-phosphate
PEP: Phosphoenolpyruvate
HRMS: High-resolution mass spectrometry

## References

[1] Hugenholtz J, Smid EJ (2002). Nutraceutical production with food-grade microorganisms. Curr Opin Biotechnol.

[2] Wang Y, Liu L, Jin Z, Zhang D (2021). Microbial cell factories for green production of vitamins. Front Bioeng Biotechnol.

[3] Pappenberger G, Hohmann H-P (2013). Industrial production of L-ascorbic acid (vitamin C) and D-isoascorbic acid. Biotechnol Food Feed Addit.

[4] Averianova LA, Balabanova LA, Son OM, Podvolotskaya AB, Tekutyeva LA (2020). Production of vitamin B2 (riboflavin) by microorganisms an overview. Front Bioeng Biotechnol.

[5] Fang H, Kang J, Zhang D (2017). Microbial production of vitamin B12: a review and future perspectives. Microb Cell Fact.

[6] Kang M-J, Baek K-R, Lee Y-R, Kim G-H, Seo S-O (2022). Production of vitamin k by wild-type and engineered microorganisms. Microorganisms.

[7] Revuelta JL, Serrano-Amatriain C, Ledesma-Amaro R, Jiménez A (2018). Formation of folates by microorganisms: towards the biotechnological production of this vitamin. Appl Microbiol Biotechnol.

[8] Bailey LB, Caudill MA, Erdman JW, Macdonald IA, Zeisel SH (2021). Folate. Present knowledge in nutrition.

[9] Jägerstad M (2012). Folic acid fortification prevents neural tube defects and may also reduce cancer risks. Acta Paediatr.

[10] Ohrvik VE, Witthoft CM (2011). Human folate bioavailability. Nutrients.

[11] Saini RK, Nile SH, Keum YS (2016). Folates: chemistry, analysis, occurrence, biofortification and bioavailability. Food Res Int.

[12] Choi JH, Yates Z, Veysey M, Heo YR, Lucock M (2014). contemporary issues surrounding folic acid fortification initiatives. Prev Nutr Food Sci.

[13] LeBlanc JG, de Giori GS, Smid EJ, Hugenholtz J, Sesma F (2007). Folate production by lactic acid bacteria and other food-grade microorganisms. Commun Current Res Edu Top Trends Appl Microbiol.

[14] Saeed AH, Salam AI (2013). Current limitations and challenges with lactic acid bacteria: a review. Food Nutr Sci.

[15] Zhu L, Wang J, Xu S, Shi G (2021). Improved aromatic alcohol production by strengthening the shikimate pathway in *Saccharomyces cerevisiae*. Process Biochem.

[16] Curran KA, Leavitt JM, Karim AS, Alper HS (2013). Metabolic engineering of muconic acid production in *Saccharomyces cerevisiae*. Metab Eng.

[17] Hjortmo S, Patring J, Jastrebova J, Andlid T (2005). Inherent biodiversity of folate content and composition in yeasts. Trends Food Sci Technol.

[18] Su YK, Willis LB, Jeffries TW (2015). Effects of aeration on growth, ethanol and polyol accumulation by *Spathaspora passalidarum* NRRL Y-27907 and *Scheffersomyces stipitis* NRRL Y-7124. Biotechnol Bioeng.

[19] Jeffries TW, Van Vleet JRH (2009). *Pichia stipitis* genomics, transcriptomics, and gene clusters. FEMS Yeast Res.

[20] Shin M, Kim J-w, Ye S, Kim S, Jeong D, Lee DY (2019). Comparative global metabolite profiling of xylose-fermenting *Saccharomyces cerevisiae* SR8 and *Scheffersomyces stipitis*. Appl microbiol Biotechnol.

[21] Silva JPA, Mussatto SI, Roberto IC, Teixeira JA (2012). Fermentation medium and oxygen transfer conditions that maximize the xylose conversion to ethanol by *Pichia stipitis*. Renew Energy.

[22] Li C, Xia J-Y, Chu J, Wang Y-H, Zhuang Y-P, Zhang S-L (2013). CFD analysis of the turbulent flow in baffled shake flasks. Biochem Eng J.

[23] Agbogbo FK, Coward-Kelly G, Torry-Smith M, Wenger KS (2006). Fermentation of glucose/xylose mixtures using *Pichia stipitis*. Process Biochem.

[24] Van Maris AJ, Abbott DA, Bellissimi E, van den Brink J, Kuyper M, Luttik MA (2006). Alcoholic fermentation of carbon sources in biomass hydrolysates by *Saccharomyces cerevisiae*: current status. Antonie Van Leeuwenhoek.

[25] Grohmann K, Bothast R (1994). Pectin-rich residues generated by processing of citrus fruits, apples, and sugar beets: enzymatic hydrolysis and biological conversion to value-added products.

[26] Kilian S, Van Uden N (1988). Transport of xylose and glucose in the xylose-fermenting yeast *Pichia stipitis*. Appl Microbiol Biotechnol.

[27] Yamakawa CK, Kastell L, Mahler MR, Martinez JL, Mussatto SI (2020). Exploiting new biorefinery models using non-conventional yeasts and their implications for sustainability. Biores Technol.

[28] Liang M, He QP, Wang J (2014). Understanding xylose metabolism of *Scheffersomyces stipitis* through a central carbon metabolic network model. Adv Chem Eng Res.

[29] Hilliard M, Damiani A, He QP, Jeffries T, Wang J (2018). Elucidating redox balance shift in *Scheffersomyces stipitis*’ fermentative metabolism using a modified genome-scale metabolic model. Microb Cell Fact.

[30] Blank LM, Lehmbeck F, Sauer U (2005). Metabolic-flux and network analysis in fourteen hemiascomycetous yeasts. FEMS Yeast Res.

[31] Papini M, Nookaew I, Uhlén M, Nielsen J (2012). *Scheffersomyces stipitis*: a comparative systems biology study with the crabtree positive yeast *Saccharomyces cerevisiae*. Microb Cell Fact.

[32] Fiaux J, Çakar ZP, Sonderegger M, Wüthrich K, Szyperski T, Sauer U (2003). Metabolic-flux profiling of the yeasts *Saccharomyces*
*cerevisiae* and *Pichia*
*stipitis*. Eukaryot Cell.

[33] Gao M, Cao M, Suástegui M, Walker J, Rodriguez Quiroz N, Wu Y (2017). Innovating a nonconventional yeast platform for producing shikimate as the building block of high-value aromatics. ACS Synth Biol.

[34] Cueto-Rojas HF, Milne N, van Helmond W, Pieterse MM, van Maris AJ, Daran J-M (2017). Membrane potential independent transport of NH3 in the absence of ammonium permeases in *Saccharomyces cerevisiae*. BMC Syst Biol.

[35] Kanehisa M, Goto S (2000). KEGG: kyoto encyclopedia of genes and genomes. Nucleic Acids Res.

[36] Sibirny A (2019). Non-conventional yeasts: from basic research to application.

[37] Caspi R, Billington R, Fulcher CA, Keseler IM, Kothari A, Krummenacker M (2018). The MetaCyc database of metabolic pathways and enzymes. Nucleic Acids Res.

[38] da Cruz SH, Cilli EM, Ernandes JR (2002). Structural complexity of the nitrogen source and influence on yeast growth and fermentation. J Inst Brew.

[39] Hjortmo S, Patring J, Andlid T (2008). Growth rate and medium composition strongly affect folate content in *Saccharomyces cerevisiae*. Int J Food Microbiol.

[40] Serrano-Amatriain C, Ledesma-Amaro R, López-Nicolás R, Ros G, Jiménez A, Revuelta JL (2016). Folic acid production by engineered *Ashbya gossypii*. Metab Eng.

[41] Jach ME, Sajnaga E, Janeczko M, Juda M, Kochanowicz E, Baj T (2021). Production of enriched in B vitamins biomass of *Yarrowia lipolytica* grown in biofuel waste. Saudi J Biol Sci.

[42] Zhu T, Koepsel R, Domach M, Ataai M (2003). Metabolic engineering of folic acid production.

[43] Sybesma W, Starrenburg M, Tijsseling L, Hoefnagel MH, Hugenholtz J (2003). Effects of cultivation conditions on folate production by lactic acid bacteria. Appl Environ Microbiol.

[44] Verduyn C, Postma E, Scheffers WA, Van Dijken JP (1992). Effect of benzoic acid on metabolic fluxes in yeasts: a continuous-culture study on the regulation of respiration and alcoholic fermentation. Yeast.

[45] Patring JD, Jastrebova JA, Hjortmo SB, Andlid TA, Jägerstad IM (2005). Development of a simplified method for the determination of folates in baker's yeast by HPLC with ultraviolet and fluorescence detection. J Agric Food Chem.

