# Supplementary material for: First report on Vitamin B9 production including quantitative analysis of its vitamers in the yeast Scheffersomyces stipitis
Source: Biotechnol Biofuels Bioprod. 2022 Sep 19;15:98. doi: 10.1186/s13068-022-02194-y (PMC9487109; doi:10.1186/s13068-022-02194-y)
Supplement: Supplementary file 2 — Additional file 2: Supplementary figures. Figure S1. Fermentation profile on (A) Verduyn-S 20 g/L glucose m/f 1:5 and (B) Verduyn-S 20 g/L glucose in baffled flasks. Figure S2. Fermentation profile on (A) Verduyn-S2 20 g/L xylose in baffled flasks. Figure S3. Fermentation profile on (A) Verduyn 20 g/L glucose + 10 g/L xylose in baffled flasks and (B) Verduyn-S2 20 g/L glucose + 10 g/L xylose in baffled flasks. Figure S4. Cofactor imbalance for xylose assimilation under different oxygen conditions. Figure S5. EIC (Extracted Ion Chromatogram) in negative ion current of a representative sample (in brown) showing the occurrence of the three reduced vitamers and the absence of folic acid compared with the reference standards (in black). [file 13068_2022_2194_MOESM2_ESM.docx]

**Supplementary material**

**Figure S1**


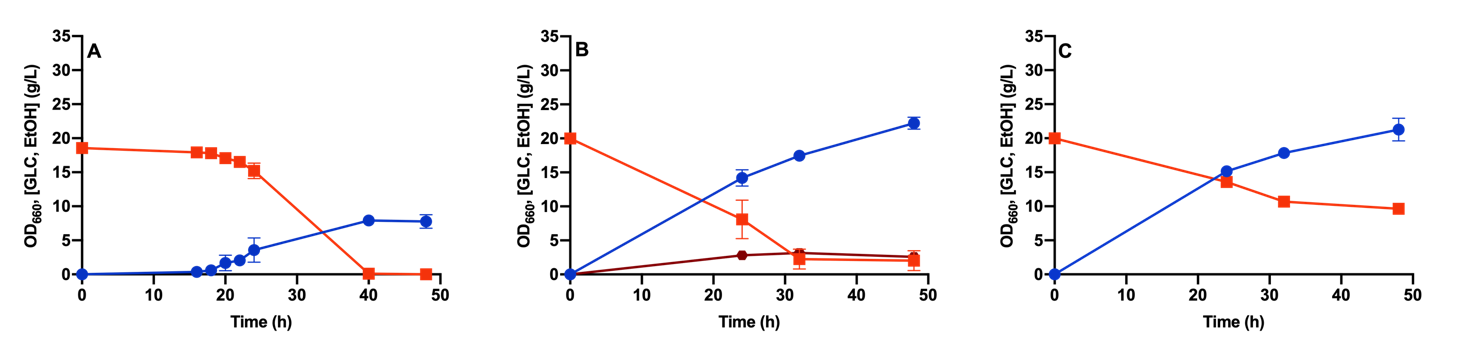


The figure shows the fermentation profile on **(A)** Verduyn-S 20g/L glucose m/f 1:5 and **(B)** Verduyn-S 20g/L glucose in baffled flasks. Figure key: OD (full blue circles), glucose consumption (full orange squares), ethanol production (dark brown hexagons).

**FIgure S2**


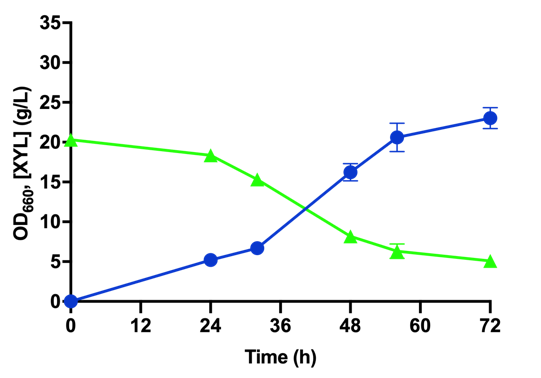


The figure shows the fermentation profile on **(A)** Verduyn-S2 20 g/L xylose in baffled flasks. Figure key: OD (full blue circles), xylose consumption (full green triangles).

**Figure S3**


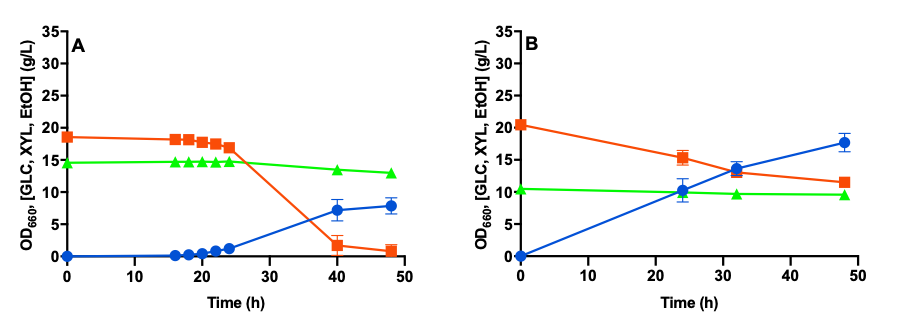


The figure shows the fermentation profile on **(A)** Verduyn 20g/L glucose + 10 g/L xylose in baffled flasks and **(B)** Verduyn-S2 20g/L glucose + 10 g/L xylose in baffled flasks. Figure key: OD (full blue circles), glucose consumption (full orange squares), xylose consumption (full green triangles).

In Verduyn medium, glucose was consumed within 50 h, the final OD reached was 7.9, with a biomass yield of 0.41 OD/g; xylose consumption was observed only after glucose depletion, due to glucose catabolite repression mechanism; however, *S. stipitis* was able to consume only 1.5 g/L of xylose (**Figure S3, A**). Accordingly, the growth profile and biomass yield are very similar to those observed on glucose alone (**Figure 1A**).

Given the positive effect of high oxygenation and the results obtained on individual sugars, the kinetics were repeated in baffled flasks (**Figure S3, B**). The expected result was the rapid consumption of 10 g/L of glucose, followed by a slowdown in growth, as observed previously. The observed behavior, however, is again unexpected: *S. stipitis* did consume 10 g/L of glucose, but it exhibited a much slower growth, while achieving a high biomass titer and yield (20.1 OD; 1.56 OD/g). This behavior is similar to the one observed in the presence of xylose 20 g/L alone (**Figure 1B**).

**Figure S4**

**
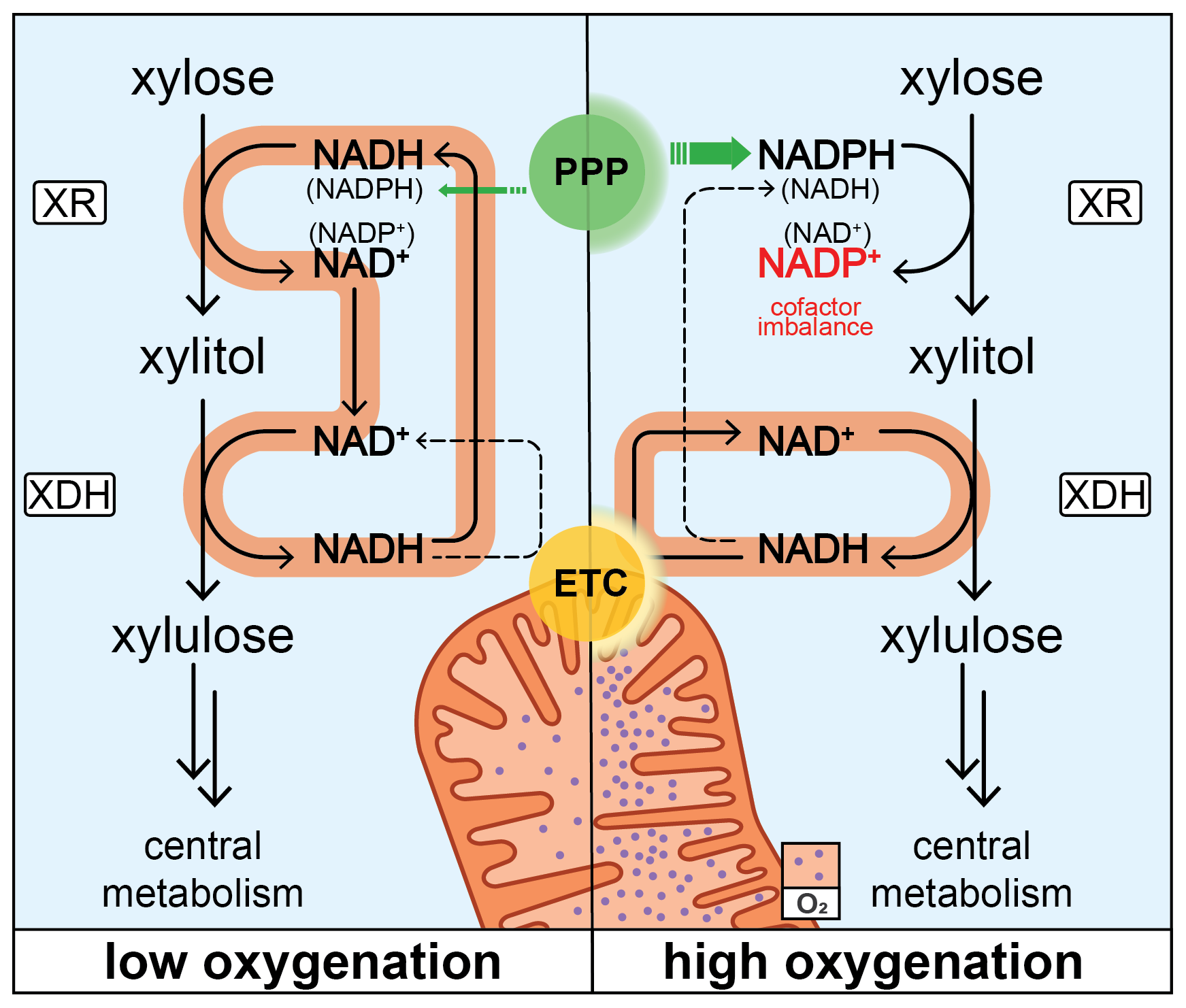
**

Cofactor imbalance for xylose assimilation under different oxygen conditions.

The different energetics of the uptake of ammonium sulfate and urea partially explain our observations regarding fermentation profiles under different oxygenation. It is necessary to consider that nitrogen metabolism is strictly regulated by the Nitrogen Catabolite Repression (1) and that in *S. cerevisiae* it has been observed that the gene expression pattern is different when the available nitrogen source is ammonium or urea (2).This implies that the higher consumption of xylose is probably due to other, more complex regulatory phenomena as well.

Indeed, these observations are not sufficient to explain why this behavior occurs only in the presence of high concentrations of xylose, and not in the other conditions that were tested. While glucose requires two phosphorylation steps to be metabolized, xylose requires a reduction to xylitol (by xylose reductase, XR) and a subsequent oxidation to xylulose (by xylose dehydrogenase, XDH). The XR requires NAD(P)H as cofactor, while the XDH cofactor is only NAD^+^ dependent. Interestingly, the specificity of XR for NADH is greater when growth is limited by oxygen, as NADH cannot be readily re-oxidized on the electron transport chain; when oxygen uptake is sufficient, NADH is less available and causes a specificity shift of XR towards NADPH, with a consequent increase in flow through the PPP (3, 4). These observations could justify the different behavior observed in the presence of xylose or glucose, due to a possible imbalance of the redox cofactors in the presence of high concentrations of xylose and oxygen.

**Figure S5**
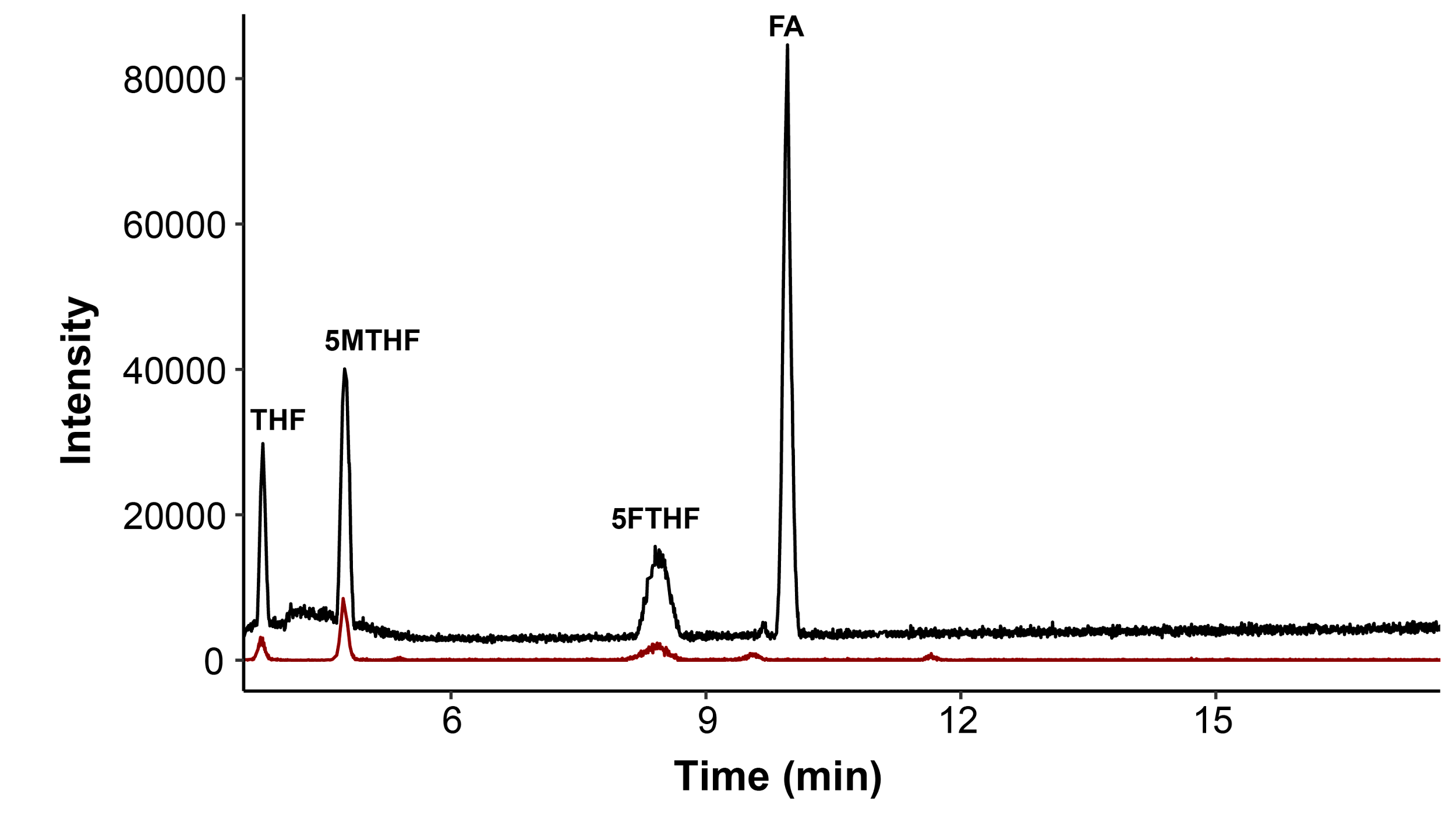


EIC (Extracted Ion Chromatogram) in negative ion current of a representative sample (in brown) showing the occurrence of the three reduced vitamers and the absence of folic acid compared with the reference standards (in black).

1. da Cruz SH, Cilli EM, Ernandes JR. Structural complexity of the nitrogen source and influence on yeast growth and fermentation. Journal of the Institute of Brewing. 2002;108(1):54-61.

2. Godard P, Urrestarazu A, Vissers S, Kontos K, Bontempi G, van Helden J, et al. Effect of 21 different nitrogen sources on global gene expression in the yeast Saccharomyces cerevisiae. Molecular and cellular biology. 2007;27(8):3065-86.

3. Shin M, Kim J-w, Ye S, Kim S, Jeong D, Lee DY, et al. Comparative global metabolite profiling of xylose-fermenting Saccharomyces cerevisiae SR8 and Scheffersomyces stipitis. Applied microbiology and biotechnology. 2019;103(13):5435-46.

4. Hilliard M, Damiani A, He QP, Jeffries T, Wang J. Elucidating redox balance shift in Scheffersomyces stipitis’ fermentative metabolism using a modified genome-scale metabolic model. Microbial cell factories. 2018;17(1):1-15.
